# Biosensors based on novel nonlinear delta-function photonic crystals comprising weak nonlinearities

**DOI:** 10.1038/s41598-022-22210-3

**Published:** 2022-10-21

**Authors:** Ahmed Mehaney, Mazen M. Abadla, Hussein A. Elsayed

**Affiliations:** 1 TH-PPM Group, Physics Department, Faculty of Science, Beni-Suef University, Beni Suef, 62512 Egypt; 2 Physics Department, Faculty of Science, Al-Aqsa University, Gaza, Palestinian Authority Palestine

**Keywords:** Biophysics, Computational biology and bioinformatics, Engineering, Materials science, Mathematics and computing, Optics and photonics, Physics

## Abstract

In this research, we propose a novel nonlinear delta-function photonic crystal for detecting sodium iodide (NaI) solution of different concentrations. The suggested structure comprises 50 delta stacks of GaP in an aqueous solution of NaI. These stacks are considered to have weak defocusing nonlinearity in the order of 10^−6^ (V/m)^−2^. Due to nonlinearity of the design, a defect-like resonance is formed within the photonic band gap. Thus, the detection of NaI with different concentrations can be easily investigated without the inclusion of a defect through the photonic crystal structure. The effects of both the linear part of the refractive index of GaP layers and nonlinear coefficient on the transmittance value are thoroughly discussed. The numerical findings investigate that the resonant peak begins to split at some critical nonlinearity. In our proposed structure, splitting occurs at about − 12 × 10^−6^ (V/m)^−2^. In this regard, the suggested sensor provides a high sensitivity of 409.7 nm/RIU and a wonderful detection limit of 0.0008.

## Introduction

In recent years, an increasing interest in the study of wave propagation through periodic structures, especially one-dimensional (1D) designs, have been demonstrated. Amongst these structures, the most important one is the photonic crystal (PC) or photonic band gap (PBG) material, which was first introduced by Yablonovitch and John^1,2^. Following that, PCs were fabricated in 1D, 2D and 3D structures for various applications^3–6^. These crystals are periodically modulated nanostructured materials accomplishing multiple interferences of the incident waves at each interface of these materials^5,6^. Physically, the interface forms due to the difference in the dielectric constant of each material, similar to the difference in the Fermi level of electronic band gap and semiconductor materials^1,7,8^. Based on the optical mismatch between the constituents of PCs, PBGs may be introduced. This PBG leads to some new physical properties and numerous potential applications that cannot be investigated using the conventional materials^9,10^.

The aim of this research is to study the electromagnetic wave propagation through a delta function 1D PC composed of delta stacks (nonlinear (NL) material) of gallium phosphide (GaP) and located in an aqueous solution for sensing purposes. The heterostructure of the PC is relevant to the Kronig-Penney model that describes the electron motion in a 1D periodic potential barrier^11,12^. It is well-known that disordered structures in linear materials induce a localizing effect for the propagated wave while the nonlinear interaction introduces a type of delocalization effect for the incident wave through periodic systems^13^. In disordered one-dimensional lattices, Anderson's theory predicts an exponentially decaying transmittance with the structure length^14^. However, the transmission coefficient has been proven to decay slowly in nonlinear structures^15,16^. On the other hand, it is worth mentioning that the nonlinear interactions introduce some disorder in the periodic structure and hence enhance the localization effect for the incident wave^12,13^. Some studies confirm that for large nonlinearities, the delocalization effect of the propagated wave disappears distinctly^17^. Motivated by the aforementioned works, we intend here to enhance the localization effect of the incident electromagnetic waves in the visible region through a nonlinear delta function PC by inserting the design in an aqueous solution (liquids are unique for wave localization and resonance effects^18,19^ and as well by increasing the nonlinearity in the PC design. Consequently, the second purpose of the research here is to study the effects of the nonlinearity on the transmission spectra of the PC and show how the transmission coefficient decays by defocusing the nonlinearity of the proposed nonlinear materials.

It should be mentioned that the wave propagation in nonlinear systems has been extensively researched for the sake of understanding optical phenomena and electronic transport properties of many systems such as superlattices^20^ and Nano-devices^21^. The non-linear Schrodinger equation is considered the prototype for different nonlinear physical phenomena^22^. In a superfluid, for example, it is related to the Gross–Pitaevsky equation whereas in an electronic system it is related to the Coulomb interactions between confined electrons^20,22^. For the above-mentioned reasons, it is clear that the transmission of the incident waves is not uniquely determined for linear and nonlinear systems.

The transfer matrix method (TMM) is considered as an accurate method for manipulating wave propagation through layered media^3,6,9^. Besides, it is intensively used to describe wave propagation through 1D PCs^3,6,9^, distributed feedback lasers^23^ and uniform and non-uniform gratings^24^. Moreover, some studies discussed the possible usage of this tool in nonlinear optical systems^24^. Since, the TMM has been used to analyze the 1D Schrodinger equation in an arbitrary quantum well design^15^ and solving of tunneling in superlattices^24^. Although a bit modified, the method developed here is based on the ordinary TTM, commonly employed to calculate different photonic band structures.

For the reasons mentioned above, PC sensors have been widely used in many academic and industrial applications as they provide novel results and high performance^3,9,19^. Among them also are the PC micro cavities^25^, slot PC waveguides^26–31^, slab waveguides^32^, ring-shaped slotted PC waveguides^33^ and Nano-cavities^34–39^. Motivated by the aforementioned works, this work focuses on studying a new type of PC sensors based on a Kronig–Penney delta function (nonlinear materials) PC. In spite of most of the previous PC designs provided high Q factor and were used for various liquids and gases applications, the presented sensor has an obvious difference from the conventional PC sensors as it does not depend on the defect modes or waveguides that suffer from some drawbacks such as high dissipation for the propagated wave and the wave signal may shrink through the detection process^40^. In addition, another main advantage of utilizing a nonlinear structure is that its nonlinear response is ultra-rapid response. Moreover, the weak nonlinearity of the material used here considered another solution for the problem of the delocalization effect that occurs in large nonlinear structures. Therefore, we will also study the effects of defocusing nonlinearity of the used material on the intensity of the transmission coefficient. In addition, the suggested design may provide some facilities in the diffusion of the analyse compared to the aforementioned designs. Furthermore, the suggested design could be simply fabricated based on the chemical wet etching approach that was widely considered in the fabrication of PCs^41^. Thus, we believe that such advantages could be valuable regarding the real environment.

On the other hand, the liquid of interest here is the sodium iodide (NaI) which is used in the treatment of some physiological disorders such as thyroid disorder besides the various medical and industrial applications^42^. For example, NaI water mixtures are used to treat iodine deficiency caused by poor nutrition^42^. Based on the promising points stated-above, we intend here in this research to exploit the merits of nonlinear structures in the introduction of a highly sensitive biosensor by using a 1D nonlinear Delta-function PC**.** The research is classified into the following sections. The theoretical analysis of the Kronig–Penny NL delta function PC and the mathematical equations of the transmission spectra of the incident electromagnetic wave are introduced in “Model description and mathematical analysis” section. In “Numerical demonstrations and discussion” section, numerical results and discussions about the effects of non-linearity, refractive index of non-linear material, angle of incidence, NaI concentrations, and other parameters on the transmission coefficient are presented. Finally, conclusion remarks are made in “Conclusion” section.

## Model description and mathematical analysis

### Wave propagation in NL structures

**Figure 1:**
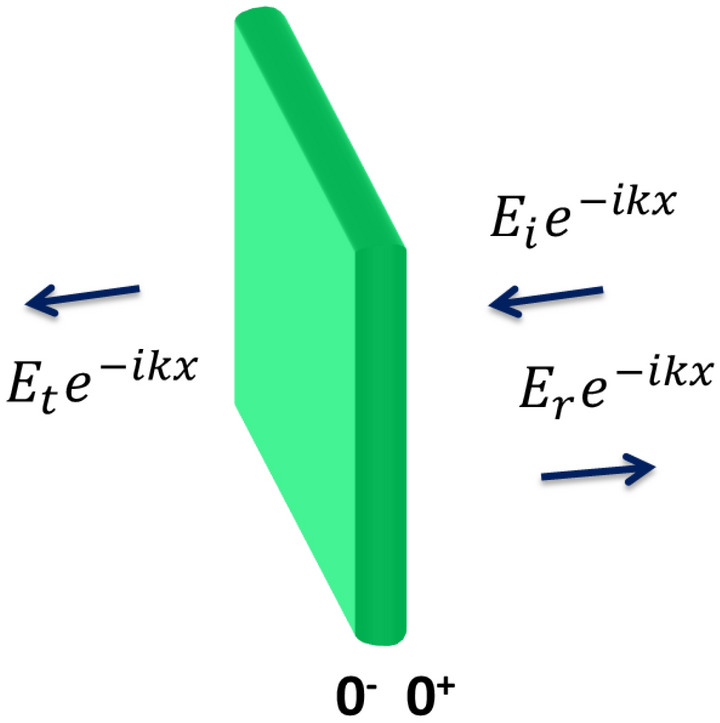
A single non-linear delta function layer.

As seen in Fig. 1, we first study a single very thin NL delta ($\delta$) function layer of permittivity: $\varepsilon_{NL} \left( x \right) = U\left( {1 + {\Lambda }\left| E \right|^{2} } \right)$, where E is the electric field along the *x*- axis, $U = \varepsilon_{L} \delta x$, $\varepsilon_{L}$ is the electric permittivity and the quantity $\alpha = \varepsilon_{L} {\Lambda }$ represents the nonlinear Kerr coefficient. Focusing and defocusing NL media are described by $\alpha$ being positive or negative, respectively.

Suppose that a plane wave is incident on the NL boundary so that a part of the incident wave is reflected and the other is transmitted. In the NL medium, Maxwell's equations are given as^12,43^:

1 $$ \frac{{\partial^{2} E}}{{\partial x^{2} }} + k_{o}^{2} \varepsilon_{NL} \left( x \right)E = 0 $$

where $k_{0}$ is the wave vector of waves propagating in free space. Integrating Eq. (1) with respect to *x* would give the following equation:

2 $$ \mathop \int \limits_{{0^{ + } }}^{{0^{ - } }} \frac{{\partial^{2} E}}{{\partial x^{2} }}dx = \left. {\frac{\partial E}{{\partial x}}} \right|_{{x = 0^{ + } }} - \left. {\frac{\partial E}{{\partial x}}} \right|_{{x = 0^{ - } }} = - k_{o}^{2} \mathop \int \limits_{{0^{ - } }}^{{0^{ + } }} \varepsilon_{L} \left( {1 + {\Lambda }E^{2} } \right)\delta x Edx $$

Making use of the relation:$\mathop \int \limits_{ - a}^{ + a} \delta x f\left( x \right)dx = f\left( 0 \right)$ for any f(*x*) continuous at *x* = 0 then:

3 $$ \left. {\frac{\partial E}{{\partial x}}} \right|_{{x = 0^{ + } }} - \left. {\frac{\partial E}{{\partial x}}} \right|_{{x = 0^{ - } }} = \left. {k_{o}^{2} \varepsilon_{L} \left( {1 + {\Lambda }E^{2} } \right)E} \right|_{x = 0} $$

At x = 0, $E_{0}^{ + } = E_{0}^{ - } = E_{i} + E_{r} = E_{t}$ so that Eq. (3) can be written as:

4 $$ - ikE_{i} + ikE_{r} + ikE_{t} = k_{o}^{2} \varepsilon_{L} \left( {1 + {\Lambda }E_{t}^{2} } \right)E_{t} $$

Collecting terms and arranging then one writes:

5 $$ \left| {E_{i}^{2} } \right| = \left[ {1 + \tau \left( {1 + {\Lambda }|E_{t}^{2} |} \right)^{2} } \right] \left| {E_{t}^{2} } \right|,\,\,\tau = \left( {\frac{{k_{o}^{2} \varepsilon_{L} }}{2k}} \right)^{2} = \frac{{k_{0}^{2} \varepsilon_{L}^{2} }}{{4n^{2} }} $$

Out of this relation, transmittance is calculated as:

6 $$ T = \frac{{\left| {E_{t}^{2} } \right|}}{{\left| {E_{i}^{2} } \right|}} = \frac{1}{{1 + \tau \left( {1 + {\Lambda }|E_{t}^{2} |} \right)^{2} }} $$

In linear media, however, ${\Lambda } = 0$ and the transmittance becomes:

7 $$ T = \frac{1}{1 + \tau } = \frac{1}{{1 + \frac{{k_{0}^{2} \varepsilon_{L}^{2} }}{{4n^{2} }}}} = \frac{4}{{4 + k_{0}^{2} \varepsilon_{L}^{2} /n^{2} }} $$

### Kronig Penny multi-delta-function layers

**Figure 2:**
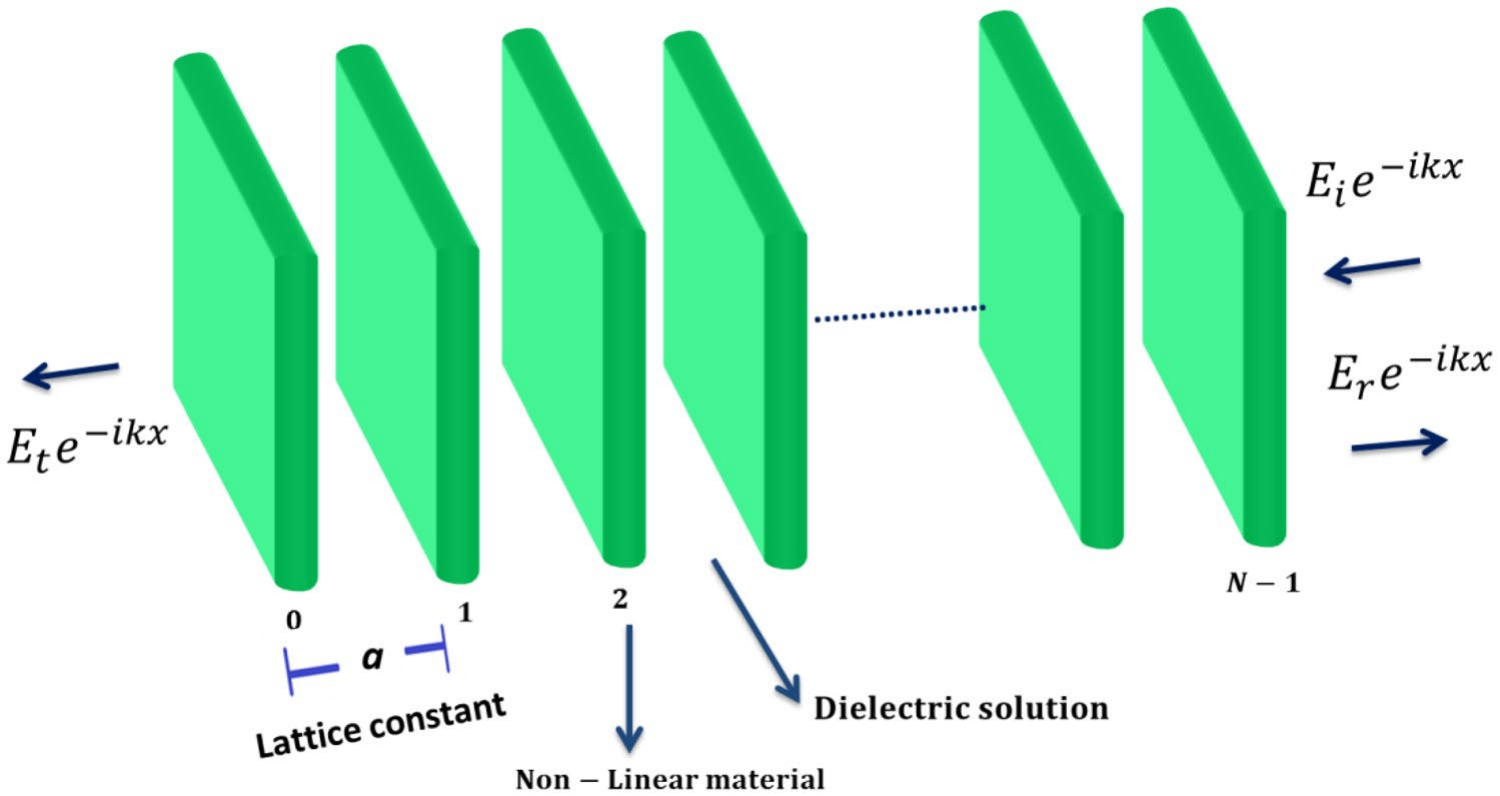
Immersion of a 1D priodic Kronig–Penny non-linear δ-function PC in an aqueous solution.

As shwon in Fig. 2, we assume a 1D priodic Kronig Penny δ-function PC multilayer (the multilayer design from the above-mentioned non-linear δ-function layer) located at *x* = *na, n* = 0,1,2…*N* − 1, and *a* is the lattice constant. The nonlinear layers are immersed in a semi-infinite dielectric medium acting as a defect or impurity in the medium.

The PC structure under consideration consists of nonlinear δ-function layers immersed in an aqueous solution as an impurity medium. The nonlinearity ${\Lambda  }$ < 0 (defocusing nonlinearity) is adopted here.

A plane wave $E_{i} e^{ - ikx}$ is incident (through air) from the right on the first NL layer. The incident, reflected and transmitted fields at the cladding layers of the PC (outside the crystal) are given as^43,44^:

8 $$ E\left( x \right) = \left\{ {\begin{array}{*{20}l} {E_{i} e^{ - ikx} + E_{r} e^{ikx} ;} \hfill & {x \ge \left( {N - 1} \right)a} \hfill \\ {E_{t} e^{ - ikx} ;} \hfill & {x \le 0} \hfill \\ \end{array} } \right. $$

Inside the NL insets, the TE-mode of the electric field satisfies the following time-independent wave equation:

9 $$ \frac{{\partial^{2} E}}{{\partial x^{2} }} + k_{o}^{2} U \mathop \sum \limits_{n = 0}^{N - 1} \left( {1 + {\Lambda }\left| E \right|^{2} } \right)\left| E \right|\left( {x - na} \right) = 0 $$

here $U = n^{2} \delta a$, n being the refractive index of the medium.

The above equation is analogous to the Kronig Penny model of electrons in 1D periodic lattices^11,12^. This equation can be rewritten in a discrete form called the generalized Poincare' map as^12,44,45^:

10 $$ E_{n + 1} + E_{n - 1} = E_{n} [2\cos k_{0} a - k_{0} U\left( {1 + {\Lambda }\left| {E_{n} } \right|^{2} } \right)\sin k_{0} a] $$

where $E_{n}$ is the electric field amplitude in the TE-polarization at the *nth* site. Equation (15) relates the amplitudes of the electric-field at three consquective locations along the *x*-axis. In numerical iterative procedre we put the initial conditions $E_{0} = 1$ and $E_{ - 1}$ = $E_{0}$ *e*^*ika*^ = *e*^*ika*^ and proceed the calculations. Thus we start with:

11 $$ E_{1} + E_{ - 1} = E_{0} [2\cos k_{0} a - k_{0} U\left( {1 + {\Lambda }\left| {E_{0} } \right|^{2} } \right)\sin k_{0} a] $$

This gives us $E_{1}$. Continuing, we get:

12 $$ E_{2} + E_{0} = E_{1} [2\cos k_{0} a - k_{0} U\left( {1 + {\Lambda }\left| {E_{1} } \right|^{2} } \right)\sin k_{0} a] $$

This gives us $E_{2}$ and so on up to the electric field at the end sites $E_{n - 1}$ and $E_{n}$. Having evaluated them one uses the equation of the transmission coefficient as:

13 $$ T = \frac{{4\sin^{2} k_{0} a \left| {E_{0} } \right|^{2} }}{{\left| {e^{{ - ik_{0} a}} E_{n} - E_{n - 1} } \right|^{2} }} $$

It is worth mentioning that the transmission (*T*) is dependent on the amplitude of the electric field at the end sites**,** $E_{n - 1}$ and $E_{n}$.

## Numerical demonstrations and discussion

In this part of our research study, we demonstrate the numerical investigation of our proposed structure in the presence of Kerr-like nonlinearity effects in the form of defocusing nonlinearity. The structure consists of 50 equally spaced layers of GaP with 3 nm thickness, and the design is immersed in an aqueous solution of different NaI concentrations. These equally spaced stacks are 1000 nm apart of each other. The delta stacks are considered to have weak defocusing nonlinearity (negative nonlinearity constant in the order of 10^−6^ (V/m)^−2^). The refractive index of GaP ranges from about 3.8 at 450 nm to about 3.3 at 650 nm and maintains a nearly zero extinction coefficient at optical frequencies^46^. In addition, the refractive index of NaI solution is found to be strongly dependent on the wavelength of the incident radiation, temperature, and its concentration^47^. At about room temperature, the refractive index of NaI solution is described (based on the quadratic fitting of the experimental data in reference^47^) as:

14 $$ n_{NaI} = 0.2425c^{2} + 0.09511c + 1.335 $$

**Figure 3:**
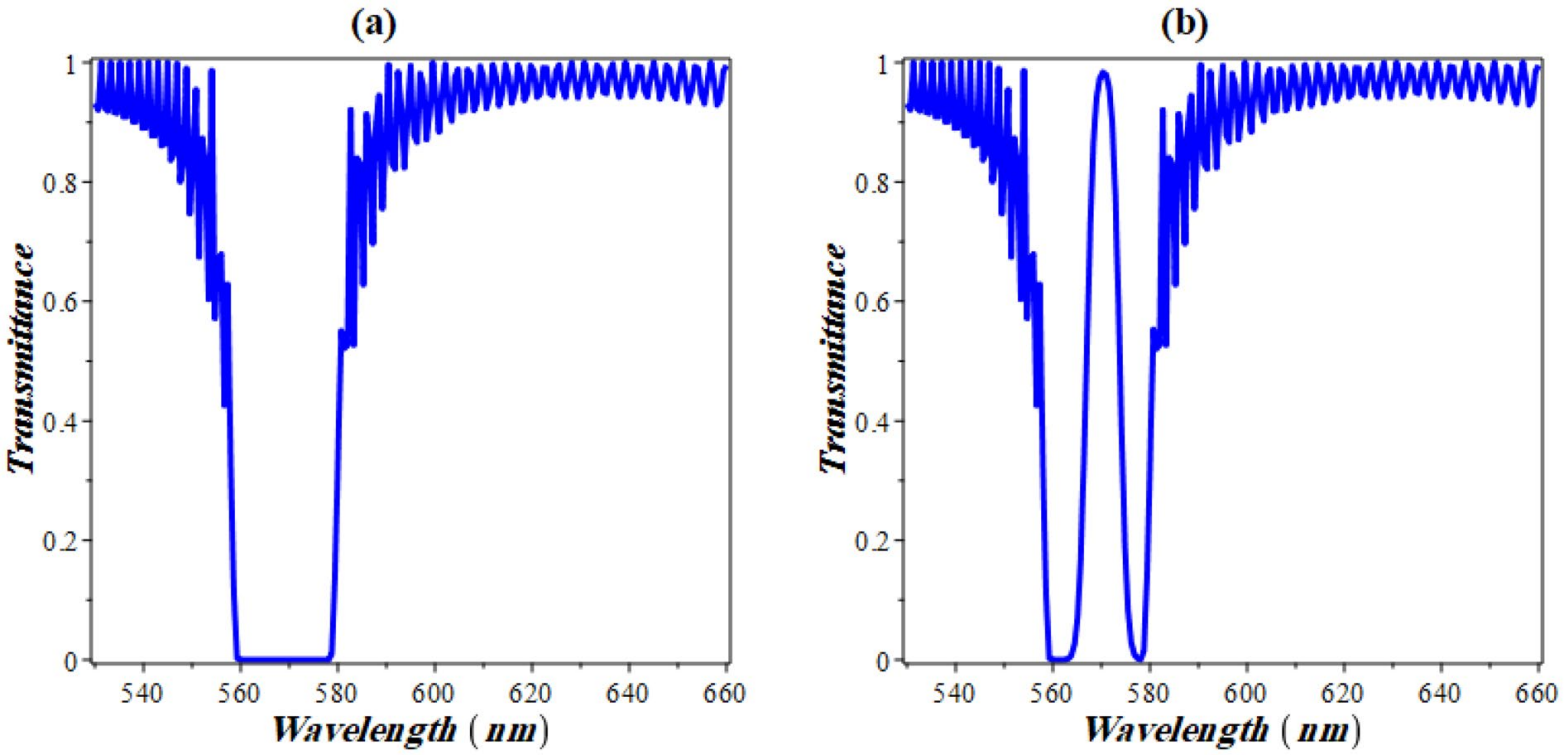
The transmittivity of the designed 1D PCs in which nonlinear stacks of GaP embedded in NaI solution with a concentration of 0.35 for: (**a**) the absence of Kerr nonlinearity and (**b**) the presence of defocusing nonlinearity.

Figure 3 demonstrates the transmittance properties of our structure. In the absence of Kerr nonlinearity, the transmittance spectrum in Fig. 3a shows the appearance of a PBG that extends between 559 and 578 nm with a bandwidth of 19 nm. This PBG is formed as a result of the relative high contrast in refractive index between GaP and NaI solution. As the defocusing nonlinearity is considered through GaP layers, the transmittance spectrum begins to take a different response due to the effect of the electromagnetic field intensity on the refractive index of GaP layers^13^. It is very interesting that even though the structure is not defected, the spectrum has been found to have a resonant peak in the center of the PBG at 570 nm as shown in Fig. 3b. This resonance peak can be also known as nonlinearity induced defect-like resonance. The presence of this peak could be of potential interest through the detection and monitoring of many liquids compared to its counterparts in defective PCs and PC. In particular, the need of a defect layer with specified and relative low thickness is not mandatory.

**Figure 4:**
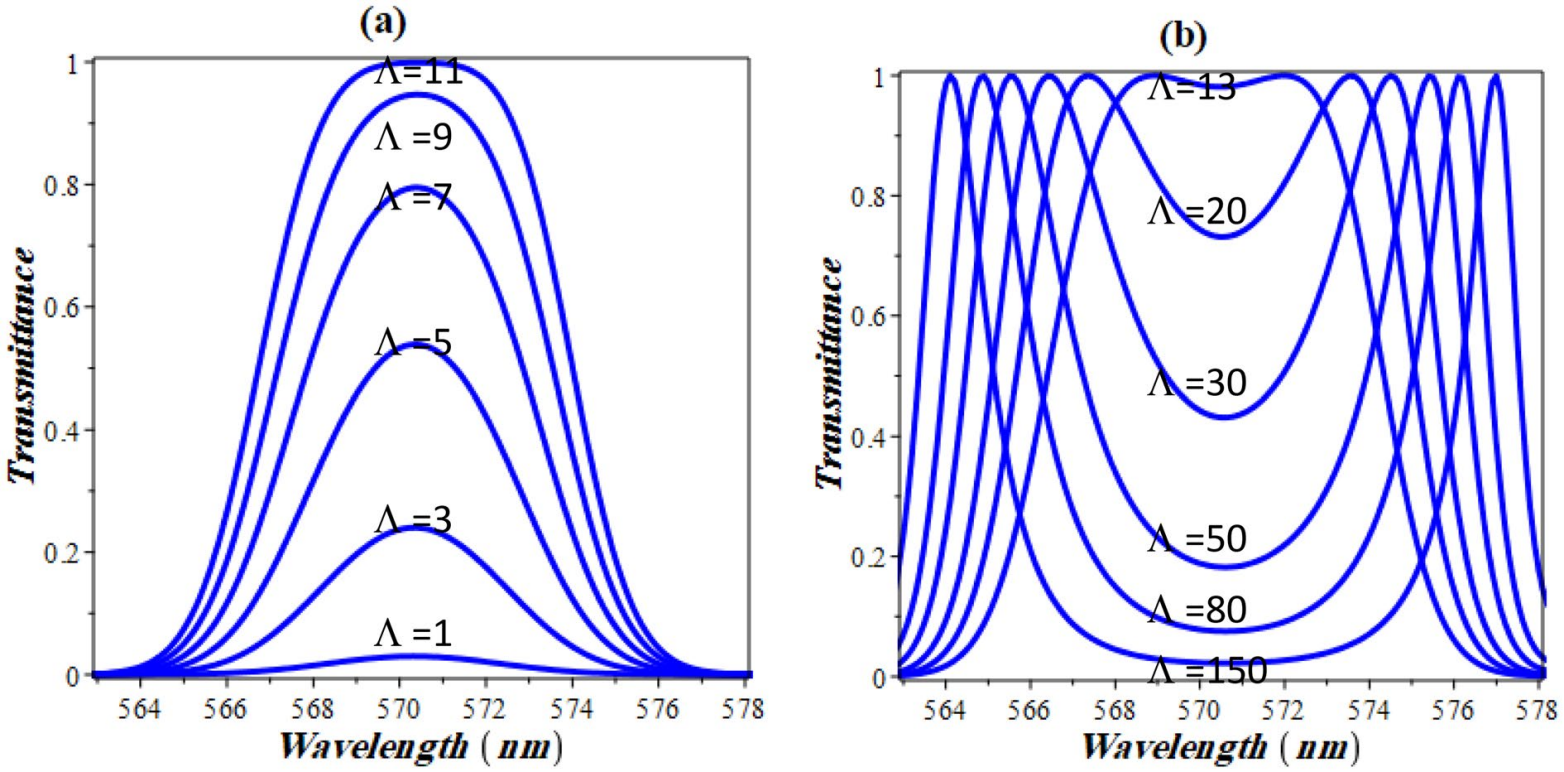
The effect of the nonlinearity coefficient on the transmittance spectrum of the designed structure for: (**a**) one defect mode like resonance and (**b**) two defect modes like resonance.

Now, we turn to show the effect of the nonlinear coefficient on the appearance of the defect like resonance peak as indicated in Fig. 4. The figure clarifies that, there exists a threshold nonlinearity coefficient after which the structure acts as if it were defected. In our structure, a nonlinearity in the order of 10^−6^ is required as shown in Fig. 4a. Once evolved, the defect-like resonance peak intensity increases with nonlinearity up to a critical value above which two peaks arise as shown in Fig. 4b. In other words, a resonant peak in the PBG evolves. The intensity now decreases with increasing the nonlinearity coefficient until a narrow sub-band gap is established. This is shown in Fig. 4b below. It can be seen from this figure that after 12 × 10^−6^ for the coefficient of nonlinearity, the peak splits into two peaks and the resonant transmittance gets reversed. As the nonlinearity increases, the two peaks diverge from each other until two distinct (well resolved) peaks with nearly no overlapping are formed. Meanwhile, the intensities of the two peaks in this case are almost equivalent to unity with the variation of the nonlinearity coefficient as shown in Fig. 4b. Therefore, within a specified value of the nonlinearity coefficient, the monitoring of NaI with different concentrations could be investigated using one or two defect modes like resonance.

**Figure 5:**
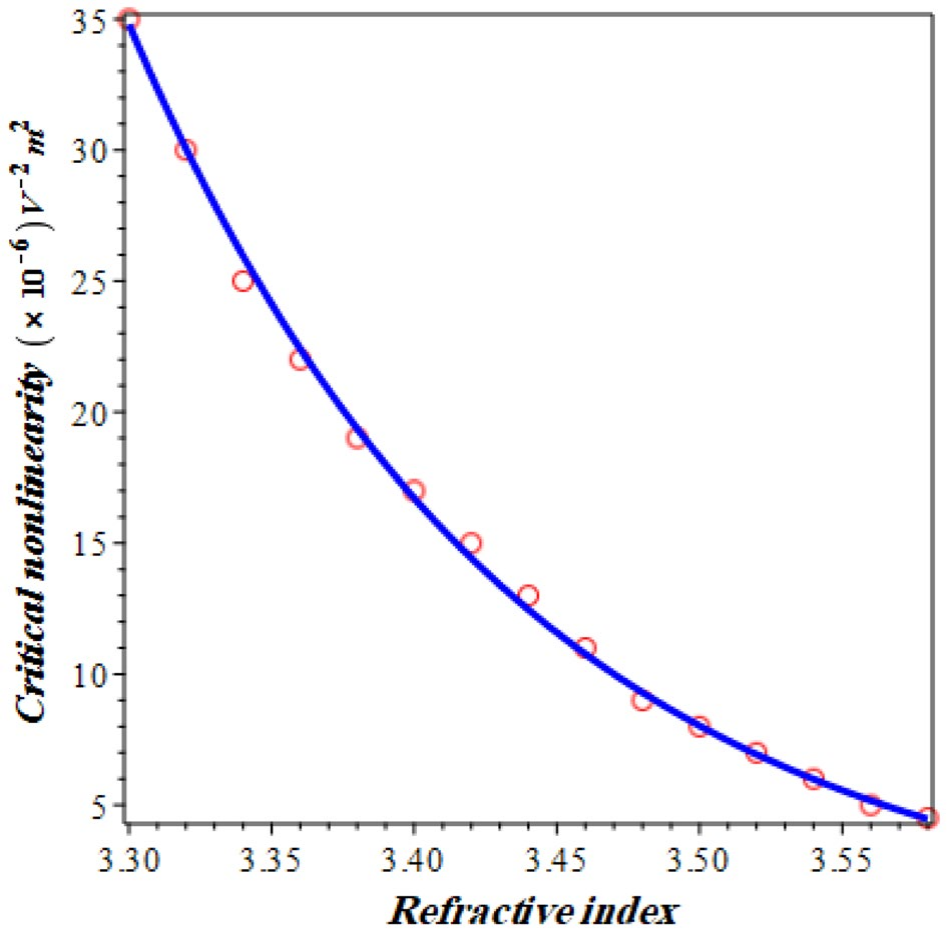
Critical nonlinearity coefficient as a function of the linear part of the delta stack refractive index.

**Figure 6:**
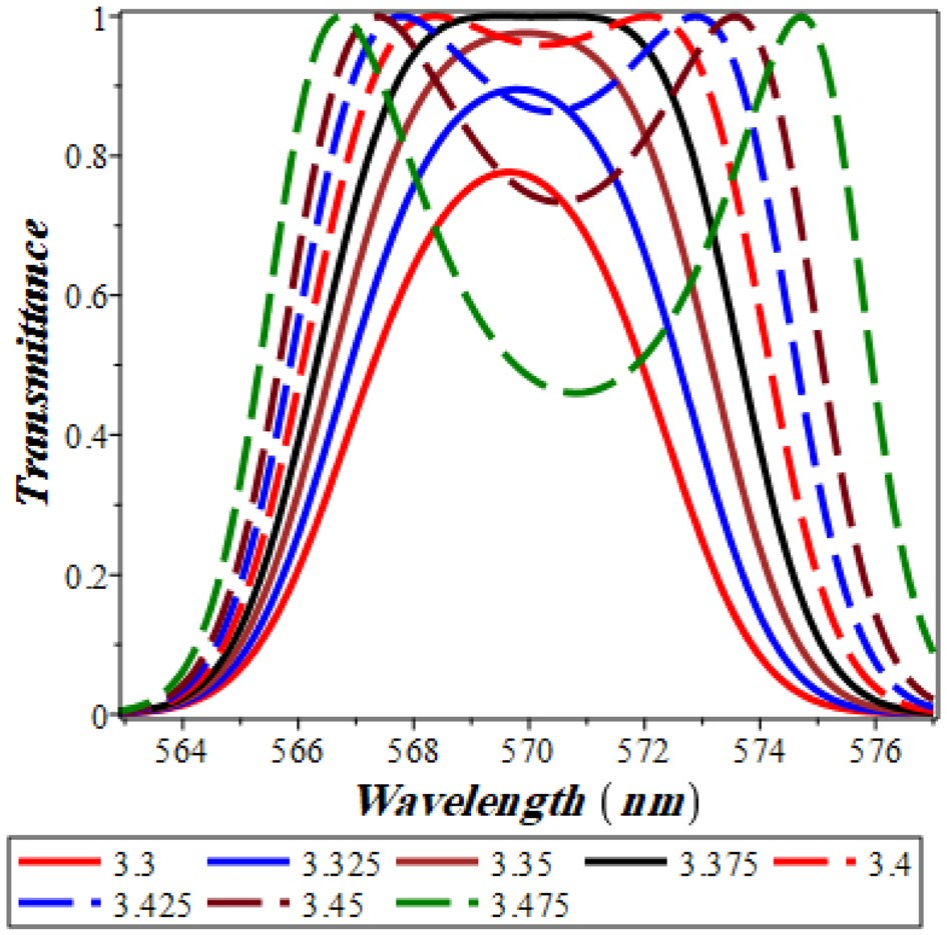
Spectrum of transmittance against the increments of the refractive index of the delta stacks at constant value of the nonlinearity coefficient = − 2 × 10^−7^(V/m)^−2^.

In Figs. 5 and 6, the dependence of the critical nonlinearity and the defect mode like resonance on the linear part of the refractive index of the delta stacks are both investigated. In particular, this visualization could be of a significant attention through the designs and fabrications of optical devices and sensors due to the role of the linear part of the refractive index in the response of the structure transmittance^13^. Figure 5 describes the variation of the critical value of the nonlinearity coefficient with the linear part of GaP layers. The figure clarifies that the critical value of Λ at which the defect mode like resonance begins to decrease exponentially with the increase of the linear part of the GaP refractive index. This response is extremely important through the detection procedure. In particular, the appearance of the resonant peak that can be used for the detection of NaI is strongly dependent on the value of the nonlinearity coefficient. In this context, the following empirical formula investigates the critical value of the nonlinearity coefficient as a function of the linear part of the refractive index in accordance with the obtained numerical results: -

15 $$ \left| {{\Lambda }_{c} } \right| = 1.21 \times 10^{6} exp\left( { - 7.332 n} \right) $$

In Fig. 6 below, the refractive index of GaP is assumed to increase from 3.3 up to 3.5 in steps of 0.025 and the corresponding transmittance is calculated. For a nonlinearity coefficient of − 20 × 10^−6^ (V/m)^−2^, a single defect like resonance is investigated as the value of delta stacks is equivalent to 3.3 at 570 nm. For further increases of the refractive index to 3.325, 3.35 and 3.375, the resonant peak is still located at the same wavelength. However, its transmittance value is significantly increasing till it reaches unity at an index of refraction = 3.375. The critical value after which peaks splitting appear is found to be 3.375 (black solid curve in Fig. 6). As the index of refraction rises above this value, the two resonant peaks become more visible and the divergence between them grows significantly, as shown in Fig. 6.

**Figure 7:**
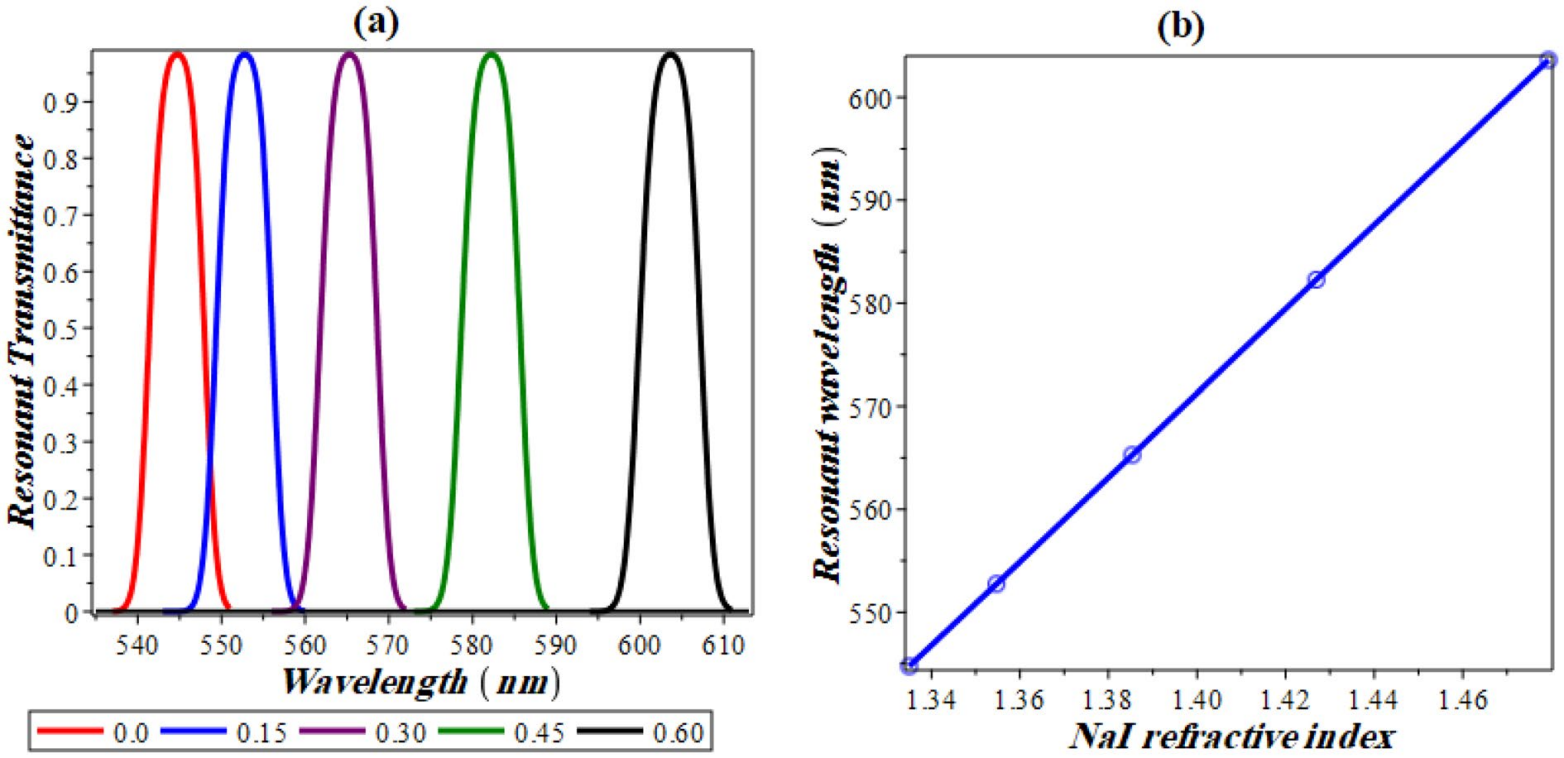
(**a**) Transmittance at resonance against wavelength for several NaI concentrations. (**b**) Resonant spectral position against concentration (or refractive index of NaI).

The transmittance characteristics of our design at different concentrations of NaI is shown in Fig. 7. Here, the value of the nonlinearity coefficient is kept constant and set to be − 10 × 10^−6^ (V/m)^−2^. This value leads to the appearance of only one defect like resonance peak as indicated in Fig. 7a. Here, the resonant wavelength is obtained at 544 nm. Such value is obtained at zero concentration of NaI through the analyte. In this regard, the position of the resonant peak is shifted towards the longer wavelengths with increasing NaI concentration as shown in Fig. 7a. Changing concentration from 0 (no NaI exists in the solution) to 0.6 results in shifting the peak from 544 to 603 nm. Accordingly, the refractive index of NaI solution changes from 1.335 up to 1.479 with respect to Eq. (14). Such change in the concentration of NaI solution results in a sensitivity value of about 409.7 nm RIU^−1^. This is considered a relative high sensitivity and has been seen in the visible region. Also, it could be improved if this design is optimized to works in IR or microwave frequencies. To sum up the response of the resonant peak against NaI concentration, we plot in Fig. 7b, the resonant wavelength against the refractive index of NaI solution. It is (completely) linear with a slope of 409.15 in an excellent agreement with our previous predictions. In particular, the slope of this relation is equivalent to the average sensitivity of our designed structure.

For further understanding of the behavior, we here calculate some performance parameters of the suggested sensor. Meanwhile, the full width at half maximum $\Delta \lambda_{1/2}$ of this structure has been calculated to be $7.39\,\,{\text{ nm}}$. Accordingly, other performance parameters can be found as the following^48–51^:

Figure of merit:

16.a $$ FOM = \frac{S}{{\Delta \lambda_{1/2} }} = \frac{408}{{7.39}} = 55.3\,\, {\text{IRU}}^{ - 1} $$

Signal to Noise Ratio:

16.b $$ SNR = \frac{{\Delta \lambda_{res} }}{{\Delta \lambda_{1/2} }} = \frac{59}{{7.39}} = 7.984 $$

Detection limit:

16.c $$ \delta n = \frac{2}{3S}\frac{{\Delta \lambda_{1/2} }}{{\left( {SNR} \right)^{0.25} }} = \frac{2}{3 \times 408.7} \frac{7.39}{{\left( {7.984} \right)^{0.25} }} = 0.007 $$

That is; this sensor can detect a minimum change of 0.007 in a refractive index unit.

**Figure 8:**
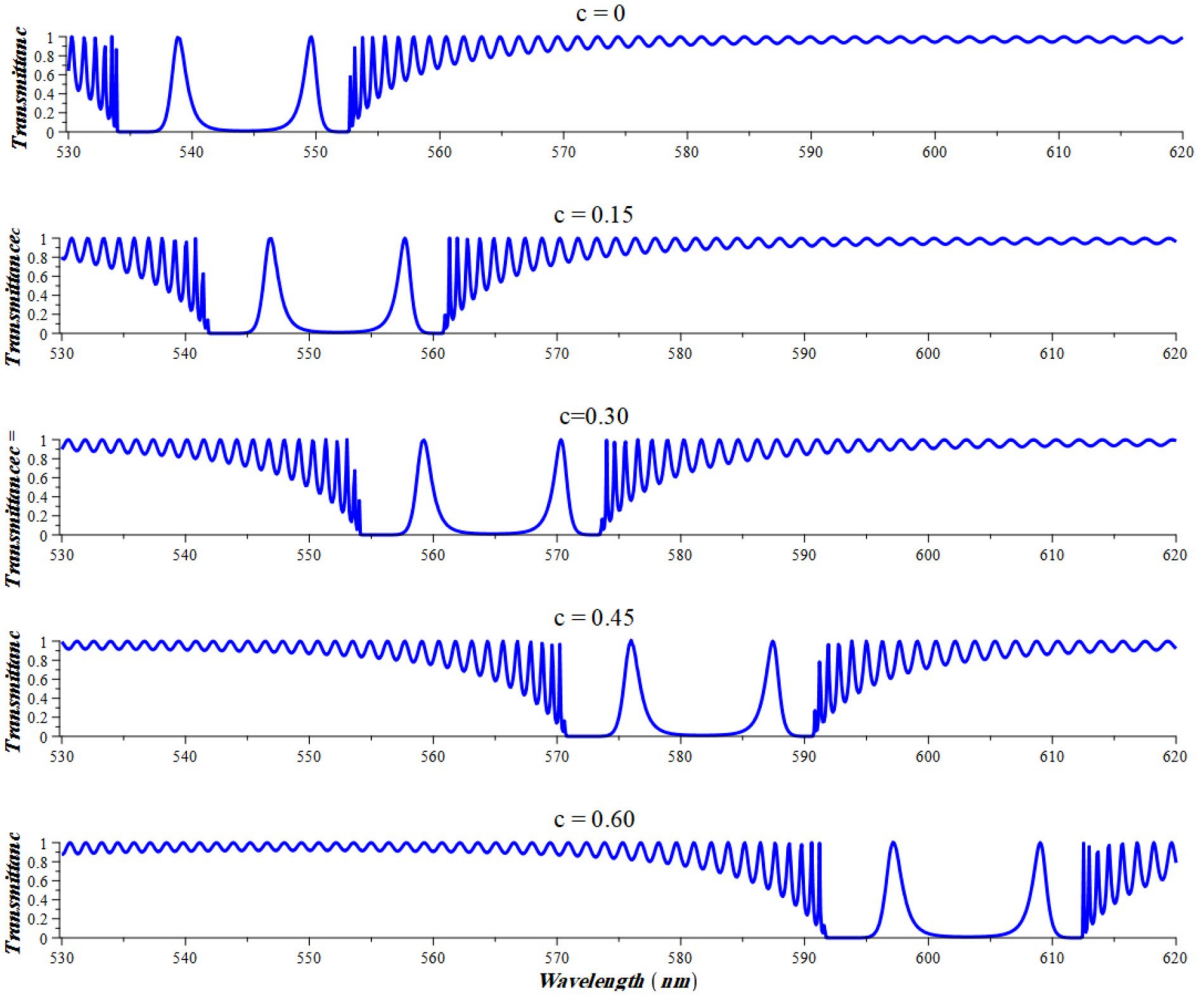
transmittance spectrum of the proposed structure operating at two-split-peaks region. N = 70, $\wedge$ = − 20 × 10^−6^ relative to the different concentrations of NaI.

Another approach of using this structure as sensor is to benefit from the two peaks split after a critical nonlinear value. Here, we choose a nonlinearity coefficient of − 20 × 10^−6^ (V/m)^−2^ and number of delta periods as N = 70 in order to have an appropriate output. As in one-peak case, the right peak shifts a spectral displacement of 59 nm subject to a change of 0.144 in refractive index as shown in Fig. 8, a case that gives sensitivity of 409.7 nm RIU^−1^. This value of sensitivity is the same as the sensitivity of the structure operating at the region of one-peak case but here the peak is much sharper, so that the performance is expected to be better. Upon calculations, the parameters related to the performance of the designed sensor have received; $$FOM = 317 \,\,{\text{RIU}}^{ - 1}$$, $$ SNR = 45.669$$, and $$\delta n = 0.0008.$$ These values show significant increments through the sensor performance compared to the case of single resonant peak. The significant increase within this parameter is due to the decrease in the value of FWHM. Finally, we have considered in Table 1 a brief comparison between the sensitivity of our proposed senor and some related ones on both theoretical and experimental levels.

**Table 1:** The performance of the proposed sensor in the vicinity of sensitivity versus some optical and photonic ones.

References	The designed structure	Sensing materials	Sensitivity (nm/RIU)
^52^	Experimental gas sensor based on TP resonance	Organic vapors	70
^53^	1D reconfigurable PCs of phase change material	Reproductive female hormones	110.67
^54^	Porous photonic crystal external cavity laser biosensor	Protein	316
^55^	Bragg-grating resonator for refractive index biosensors	Biomaterial	387.48
^56^	Four-channel label-free PC biosensor using nanocavity resonators	DNA molecule and protein	65.7
^57^	Cladding modulated grating waveguide	Biomaterial	322.96
^58^	Refractive index sensor based on Ge–Sb–Se chalcogenide microring resonator	NaCl solution	123
^59^	Fiber-optic interferometric sensor	Heavy metal ion Nickel	322
^60^	One-dimensional defective PCs	Protein	131
^61^	One-dimensional binary PCs comprising a defect layer	Hemoglobin	167
^19^	One-dimensional PCs comprising porous silicon	Soybean biodiesel	277.77
Our design	50 delta stacks of GaP separated by an aqueous solution of NaI	Sodium Iodide	409.7

## Conclusion

In summary, we have designed a 1D nonlinear delta function structure as a novel PC sensor design to detect the concentration of NaI solution based on Kerr-like nonlinearity. In this regard, the suggested structure composed of 50 delta stacks of GaP separated by an aqueous solution of NaI. The numerical results are demonstrated based on the mathematical formulism of Kronig Penny model. The numerical investigations showed the appearance of a defect like resonant peak within the PBG due to the variation of the refractive index of GaP stacks with the electromagnetic field intensity. This resonant peak could efficiently split to more than one peak with increasing the nonlinearity coefficient or the linear part of the refractive index of GaP layers. Therefore, the proposed design could be of potential concern in the detection and monitoring of NaI concentration and other chemical solutions. In this context, the suggested sensor provides a relative high sensitivity of 409.7 nm RIU^−1^ and a detection limit of 0.0008.

## Data Availability

The datasets used and/or analysed during the current study available from the corresponding author on reasonable request.
